# *Xenorhabdus* spp.: An Overview of the Useful Facets of Mutualistic Bacteria of Entomopathogenic Nematodes

**DOI:** 10.3390/life12091360

**Published:** 2022-08-31

**Authors:** Mahfouz M. M. Abd-Elgawad

**Affiliations:** Plant Pathology Department, Agricultural and Biological Research Division, National Research Centre, El-Behooth St., Dokki, Giza 12622, Egypt; mahfouzian2000@yahoo.com

**Keywords:** biocontrol, *Steinernema*, pest and pathogen management, *Xenorhabdus*, marketing

## Abstract

Mounting concern over the misuse of chemical pesticides has sparked broad interest for safe and effective alternatives to control plant pests and pathogens. *Xenorhabdus* bacteria, as pesticidal symbionts of the entomopathogenic nematodes *Steinernema* species, can contribute to this solution with a treasure trove of insecticidal compounds and an ability to suppress a variety of plant pathogens. As many challenges face sound exploitation of plant–phytonematode interactions, a full useful spectrum of such interactions should address nematicidal activity of *Xenorhabdus*. *Steinernema*–*Xenorhabdus* complex or *Xenorhabdus* individually should be involved in mechanisms underlying the favorable side of plant–nematode interactions in emerging cropping systems. Using *Xenorhabdus* bacteria should earnestly be harnessed to control not only phytonematodes, but also other plant pests and pathogens within integrated pest management plans. This review highlights the significance of fitting *Xenorhabdus*-obtained insecticidal, nematicidal, fungicidal, acaricidal, pharmaceutical, antimicrobial, and toxic compounds into existing, or arising, holistic strategies, for controlling many pests/pathogens. The widespread utilization of *Xenorhabdus* bacteria, however, has been slow-going, due to costs and some issues with their commercial processing. Yet, advances have been ongoing via further mastering of genome sequencing, discovering more of the beneficial *Xenorhabdus* species/strains, and their successful experimentations for pest control. Their documented pathogenicity to a broad range of arthropods and pathogens and versatility bode well for useful industrial products. The numerous beneficial traits of *Xenorhabdus* bacteria can facilitate their integration with other tactics for better pest/disease management programs.

## 1. Introduction

Upgrading the methods and materials used to control plant pests and pathogens via more effective and safer approaches is one of the current challenges in the field of agriculture. Growing discontent with many chemical pesticides has been elevating, due to their unacceptable impacts on human health and environmental contamination, non-target organisms, and plant-resistance development [1,2,3]. Therefore, successes of these approaches are deeply entrenched in the available opportunities to phase out such unhealthy chemicals and to displace them by friendly ecologically safe products. In this respect, challenges to traditional plant-parasitic nematode (PPN) control measures have become evident due to accumulated knowledge on plant–PPN interactions. This situation has clearly exposed the limitations of classical studies on plant–nematode interactions, and surfaced the necessity to adopt novel interdisciplinary specialty views for effective integrated pest management. Interestingly, while bacterial use has promising effects on suppressing PPNs, it enhances growth parameters and yields of the PPN-infected plants [4]. This is not to say that such traditional studies of plant–nematode interaction cannot help scientists meet challenges imposed by PPNs. I am simply arguing that these studies alone will not suffice as many interdisciplinary challenges face sound exploitation of indirect plant–nematode interactions. A full useful spectrum of such interactions should address nematicidal activity of *Xenorhabdus* (Enterobacteriales: Morganellaceae) individually or *Steinernema* (Steinernematidae: Rhabditida)–*Xenorhabdus* complex in emerging cropping systems for integrated pest management. These latter should be exploited not only for crop pest/pathogen management but also for sustainable agriculture.

Admittedly, the significance of utilizing beneficial bacteria in various programs for pest/pathogen control has been increasing. While these bacteria can substitute the toxic and frequently undesirable chemicals employed for plant protection, they can offer benign ecological technologies [4]. Among them, the genus *Xenorhabdus* comprises entomopathogenic bacterial species that can be promising alternatives for boosting the biocontrol of numerous plant pests/pathogens. These bacteria can secrete diverse arrays of operative bioactive metabolites against these pests [2,5,6,7,8,9]. Their efficacy as biopesticides is based on a massive variety of *Xenorhabdus* genes that encode for generating related compounds such as antibiotics, enzymes, bacteriocins, and toxins. They are established in the shape of pathogenicity islands on the chromosomes of different *Xenorhabdus* species.

Therefore, the current review addresses the diversity of endosymbiotic bacterial species from EPNs and their function in biological control against many plant pests and pathogens for the agro-environmental sustainability. It throws light on the recent discoveries of new species of *Xenorhabdus*, with presupposed further genes encoding for beneficial attributes that are being detected in addition to presenting genes in the *Xenorhabdus* species that are already described so that it should be further exploited or their expressed produced compounds be improved [1,8,9,10,11]. While this article offers the relevant framework of the state-of-the-art knowledge regarding *Xenorhabdus* spp., it focuses on their up-to-date biotechnology to unlock more approaches of discovery that function for boosting durable agricultural systems. This review deals with the latest discoveries on their identification, taxonomy, diversity, and utilization in the present or future management plans, especially against plant pests/pathogens.

## 2. Identification, Taxonomy, Lifestyle, and Diversity of *Xenorhabdus* spp.

### 2.1. Their Identification/Taxonomy

As *Xenorhabdus* and *Photorhabdus* bacteria (Enterobacterales: Morganellaceae) are phylogenetically close, it is not surprising that at first they were of the same genus [12]. Thus, two bacterial species in this genus (that is, *Xenorhabdus nematophila* (type species) and *X*. *luminesces*) including symbionts of the nematode genera *Steinernema* and *Heterorhabditis*, respectively, were exclusively present until 1993 [12]. Yet, the important variations in the phenotypic and molecular traits could distinguish *X*. *nematophila* from *X*. *luminesces,* leading to the transfer of all symbionts related to *Heterorhabditis* into *Photorhabdus* as a new genus with the type species *Photorhabdus luminescens* [13]. Before splitting into the two genera, some phenotypic features and symbiotic properties were utilized to characterize *Xenorhabdus* and *Photorhabdus* bacteria as two recognized groups; *P*. *luminescens* distinctly had a DNA relatedness group unlike all *Xenorhabdus* strains with significant variations in phenotypic traits [14]. *Xenorhabdus* bacteria are obviously separated from *Photorhabdus* species/strains by the 16S rDNA signature sequences [15]. Yet, both *Photorhabdus luminescens* and *X*. *nematophila* received much research work, due to being type species of their two genera, with high insecticidal activities and a global distribution.

Interestingly, the genus *Xenorhabdus* still has more homogenous species than *Photorhabdus*, but the former genus possesses a higher number of species than the latter. For example, Sajnaga and Kazimierczak [16] reported 26 *Xenorhabdus* spp. versus 19 *Photorhabdus* spp. This is possibly due to higher number of the current *Steinernema* species (the mutualistic partner of *Xenorhabdus* spp.) than *Heterorhabditis* spp. (the mutualistic partner of *Photorhabdus* spp.), i.e., >100 *Steinernema* spp. but >20 *Heterorhabditis* spp. [17]. Admittedly, there are other undescribed species related to both *Xenorhabdus* and their *Steinernema* partner which are recognized or are likely to become recognized soon. In this respect, the number of their close relatives, *Photorhabdus* species, has recently doubled, from four to twenty in the last few years [18].

Initially, three *X*. *nematophila* subspecies were raised to the species level via further description using a polyphasic technique, i.e., *X*. *poinarii*, *X. beddingi*, and *X. bovienii* [19,20]. Identification of novel *Xenorhabdus* species as EPN symbionts continued relying on sequence data of single gene sequences until the year 2007, when a compilation of 20 *Xenorhabdus* species was published [21]. Thereafter, new *Xenorhabdus* species were identified via multi-locus sequence analysis and whole genome sequence data. Some recent references, e.g., [16,22], stated that the genus *Xenorhabdus* includes the following 26 species: *X. beddingii*, *X*. *budapestensis*, *X*. *ehlersii*, *X*. *cabanillasii*, *X*. *doucetiae*, *X*. *eapokensis*, *X*. *griffiniae*, *X*. *bovienii*, *X*. *hominickii*, *X*. *innexi*, *X*. *indica*, *X*. *japonica*, *X*. *ishibashii*, *X*. *khoisanae*, *X*. *koppenhoeferi*, *X*. *magdalenensis*, *X*. *kozodoii*, *X*. *mauleonii*, *X*. *nematophila*, *X*. *miraniensis*, *X*. *poinarii*, *X*. *szentirmaii*, *X*. *romanii*, *X*. *stockiae*, *X*. *vietnamensis*, and *X*. *thuongxuanensis*. Nonetheless, other novel species are still in the pipeline. For example, *X. lircayensis* linked to its partner *S. unicornum* was recently described [23] to elevate the number to 27 species. Comprehensive biochemical, physiological, and chemotaxonomic bioassays of the bacterial strain VLS^T^ demonstrated consistent differences from the other described *Xenorhabdus* species. In addition, the authors presented phylogenetic approaches to prove that VLS^T^ is a novel strain of *X*. *lircayensis*. While they presented phylogenetic relationship placing *X*. *lircayensis* within the genus *Xenorhabdus* based on the 16S rRNA gene analysis, a phylogenetic reconstruction was also established to include *X*. *lircayensis* based on core genome sequences [23]. Ultimately, species of *Xenorhabdus* are anticipated to be raised. This expectation is based on both their importance, especially against many pests/pathogens, and the recent physiological, biochemical, and molecular techniques to identify them as mutualistic or symbiotic bacteria of *Steinernema* spp. Therefore, it could be concluded that the progressive increment in the number of novel *Xenorhabdus* species and strains and new technological methods to characterize them have led to a well-established *Xenorhabdus* taxa with finer and easier profile to study than before. Consequently, sound genome-constructed phylogenetic trees, enriched with precise sequence comparative studies, could perfectly define their diversity and relationship. These efforts for their characterization and identification should also take part in further research of the related *Xenorhabdus* spp.-bioactive complexes that can be utilized in agricultural and industrial products [2,17,24].

### 2.2. The Lifestyle and Diversity of Xenorhabdus spp.

Basically, the bacterial species in the genus *Xenorhabdus* have a mutualistic relationship with the entomopathogenic nematodes (EPNs) of the genus *Steinernema*. The bacteria live symbiotically in the specialized intestinal vesicles of *Steinernema*. The two partners naturally form an antagonist sharing mainly against their insect hosts. The EPN-third-stage infective juveniles (IJs) conserve the bacteria in their body from the outer environmental stresses until these IJs release them within the insect body. In addition, after EPN infection and depleting the host resources, the IJs vector the bacteria from one susceptible host to another. In turn, *Xenorhabdus* spp. generate antimicrobial compounds and secondary metabolites into the insect. These materials can not only kill the insect host and prepare the contents of its body to feed the nematodes for their development and reproduction, but also protect the insect cadaver from soil scavengers and saprobes [5]. During their feeding, the nematodes also swallow the bacteria in order to grow and reproduce.

Mentioning *Xenorhabdus* is sometimes linked to its counterpart *Photorhabdus* bacteria, since much similarity is generally found between them in the frame of living. *Xenorhabdus* and *Photorhabdus* comprise symbiotic bacteria of EPN-IJs, of the genera *Steinernema* and *Heterorhabditis*, respectively. Nonetheless, each of them possesses particular bacterial traits that differentiate its identity. *Xenorhabdus* spp. survive within a receptacle exhibiting biological specialization at the anterior part of the intestine. *Photorhabdus* spp. are found at the mucosa of their nematode guts [25,26]. *Steinernema* spp.-infective juveniles mature to amphimictic males and females. Therefore, the dyad (males/females) must infect their susceptible host to mate then reproduce within the insect hemocele. *Heterorhabditis*–IJs develop into hermaphrodites (females) and then, in subsequent generations, to males/females. As a result, a single hermaphroditic–IJ has the merit to infect and reproduce in the host insect. Moreover, the demeanor of bacterial colonization of *Xenorhabdus* and *Photorhabdus* differs within their nematode partners [26]. An important attribute utilized to discern the two EPN genera, after infecting their host insect, is the pigment of *Photorhabdus* (bacterium) to tinge the insect host’s body in a reddish tint. *Photorhabdus* can glow so frequently that its entire infected host usually glows in unlighted sites. To counteract the insect host-immune system, *Photorhabdus* can modify the lipopolysaccharide to tolerate the influence of the host-derived antimicrobial generation of peptides, but *Xenorhabdus* disorganizes the creation of these peptides [25]. Both genera have more than 94% identical 16S rRNA genes, but the genome may be disrupted by many inversions, translocations, deletions, and insertions [27]. *Photorhabdus* species can go through major transcriptional reformation in their host, EPN, intestine. They can stimulate general starvation mechanisms, switch to the pathway of pentose phosphate to comply with nutrition shortage and oxidative stress, cellular acidification to slacken growth, and shape biofilms to firmly last in the nematode intestine for subsequent transmission to the hemolymph of the insect [28]. These bacterial behavior can back them while lasting in the EPN gut and secure fitting transmission of the nematode–bacterium complex from one susceptible host to another. Strikingly, species of both genera are capable of growing in vitro as free-living bacteria, without their EPN partners, on artificial media with specific terms, i.e., no competition in adequate nutrient media [5,18].

It is well established that *Xenorhabdus* bacteria have essential services in the biocontrol processes against pests [5]. They, via their metabolites and exoenzymes, are in charge of the bioconversion of the infected host into nutrient soups as ideal for the development and multiplication of their nematode partner. On the other hand, recent transcriptomic analysis of both *S. carpocapsae*–*X. nematophila* and *S. puntauvense*–*X. bovienii* indicated important metabolic shifts related to the rearing conditions as well as bacterial (presence versus absence). Nematode genes involved in the production of amino acid, carbohydrate, and lipid were downregulated as IJs were cultured in vitro, in the presence or absence of the symbiotic bacteria [29]. The downregulation seems to influence the longevity of the relevant metabolism pathways. Furthermore, IJs of both *S. carpocapsae* and *S. puntauvense* displayed a unique mechanism to adjust their virulence when their symbionts were absent. Both axenic nematode species showed a differential manifestation of the toxic protein that they secrete, i.e., absence of their symbiotic bacteria raises the expression of their secreted venom protein. The study suggested that IJs of these nematode species may have a unique mechanism to adjust infecting their hosts in the absence of their *Xenorhabdus* partners [29]. This does not mean, in any way, that the fundamental role played by these mutualistic bacteria in the reproduction of nematodes and even their production on a commercial scale can be neglected or may be abandoned.

*Steinernema*–bacterial symbiont specificity and their coevolution have been thoroughly studied for many involved axenic (free of bacteria) and monoxenic (having a *Xenorhabdus* species) *Steinernema* species [16]. While a *Steinernema* species can presumably set up symbiosis with only one species of *Xenorhabdus*, any of numerous *Xenorhabdus* species are able to associate with several *Steinernema*. On the contrary, the symbiotic *Heterorhabditis*–*Photorhabdus* associations are more adaptable as many species of each partner can engage in symbiotic relationships with multiple species of the other partner [30]. These facts have recently been reviewed and backed by plenty of data [31]. However, the mechanisms underlying these relationships remain to be clarified [16]. These associations do not negate the fact that the robust specificity that favors symbionts with the most useful attributes facilitates effective transfer of such a nematode–bacterium pair from a susceptible insect pest to another. Generally, Sajnaga and Kazimierczak [16] concluded that there is a possibility of horizontal transfer of *Xenorhabdus* bacteria between different *Steinernema* species, relying on the species of *Xenorhabdus*–*Steinernema* pair used. However, such switching in the bacterium–nematode pair may have its pros and cons. On the positive side, associations of *Steinernema* species with new *Xenorhabdus* partner may validate colonization of novel niches or expand one by offering considerable fitness benefits [32,33]. These favorable results may occur when the introduced bacteria/symbiont is closely related to its native *Steinernema* species. On the negative side, the *Xenorhabdus* partner switching frequently has a harmful effect on the *Steinernema* host in terms of a reduction in their fitness, reproduction, and symbiont carriage as well as virulence. For example, *Xenorhabdus bovienii* is the native symbiotic bacteria of *S*. *feltiae*. However, using *X*. *nematophila* strain HGB315, not the native symbiont of *S*. *feltiae*, this nematode species developed and turned into gravid much faster at approximately 4 days on *X*. *bovienii* (versus 5–6 days on *X*. *nematophila* HGB315) post-seeding [34]. These detrimental outputs are usually associated with non-cognate and phylogenetically distant symbionts [16]. Eventually, researchers and stakeholders should be aware of the fact that the *Steinernema* host diversity substantially impacts coadaptation between various *Xenorhabdus*–*Steinernema* partners [35,36] for their further wise application. In this respect, Tailliez et al. [37] could identify two main groups of *Xenorhabdus* strains based on phenotypic analysis. A group included bacterial strains that can commonly grow at 35–42 °C, while the other group included *Xenorhabdus* strains that grow below 35 °C. Hence, *Xenorhabdus* bacteria may be adapted to temperate, subtropical, or tropical regions. They are also differently impacted by the growth temperature of their *Steinernema* host [37]. Moreover, the wide host range of their nematode host along with their major attributes can prove their diversity and global distribution [38] as well as give opportunities to familiarize stakeholders with the potential usage of these symbiotic bacteria [5,17]. With the global spread of the *Steinernema*–*Xenorhabdus* complex, we must mention that recent references, e.g., [18], still indicate that EPNs are not discovered in Antarctica. The long-established realization regarding the species-specific characterization for the dyad *Steinernema*–*Xenorhabdus* complex as partners for the mutualism is still effective. Thus, it can bode well for more investigations concerning their distribution in diversity and space [39]. Moreover, current research efforts have been focusing on optimizing methods and techniques for EPN surveys and extraction [15,16] to detect novel species/strains that bode well for effective and safe biocontrol of pests and pathogens and adaptation to local conditions. Therefore, it can be mostly presumed that the distribution and diversity of the EPN species is only an artefact of the linked sampling efforts [18]. However, growing interest is mainly dedicated to these bacteria when applied to suppress pests and pathogens independently, i.e., without the EPN partners [5,40,41,42,43,44].

Similar to their near relatives, *Photorhabdus* species, all the species of *Xenorhabdus* are exclusively linked symbionts to the *Steinernema* spp.–IJ stage [13]. The exception of *Photorhabdus*, materialized in *P. asymbiotica* as a human pathogen in addition to infecting insects [45], is not found in *Xenorhabdus*. None of the *Xenorhabdus* bacteria were found in free-living order in nature; therefore, they had formerly boosted doubts concerning their ability to survive and infect pathogens/pests without the EPN partner.

## 3. Pathogenicity of *Xenorhabdus* spp.

### 3.1. Magnitude and Profile of Pathogenicity

Traditionally, various *Steinernema*–*Xenorhabdus* partnerships, to attack and kill numerous arthropod pests, have been marketed and utilized as biocontrol agents [17], with increasing ambition for their expansion to prepare them for reliable alternatives in pest management and plant protection [45,46,47,48,49]. In the original status of the natural *Steinernema*–*Xenorhabdus* complex, *Xenorhabdus* host range is surely limited to the ability of the IJs to locate and infect the host. This is a prerequisite for the development and multiplication of *Xenorhabdus* spp. to achieve high levels of cells within the host. Factually, both mutualists, *Steinernema* and *Xenorhabdus*, can generate bioactive compounds to kill the invaded host [34,41]. Thereafter, the bacterial cells can modify the insect host tissues to become a nutrient diet needed for the IJ development and multiplication. Hence, the pathogenicity depends on the bacterial activity and growth. Thus, the *Xenorhabdus* rate of growth is tightly related to the time needed to kill the insect host. Clearly, *Xenorhabdus* spp. are quite virulent pathogens of a broad range of pests/pathogens including insects, fungi, bacteria, protozoa, and nematodes [41,42].

**Figure 1:**
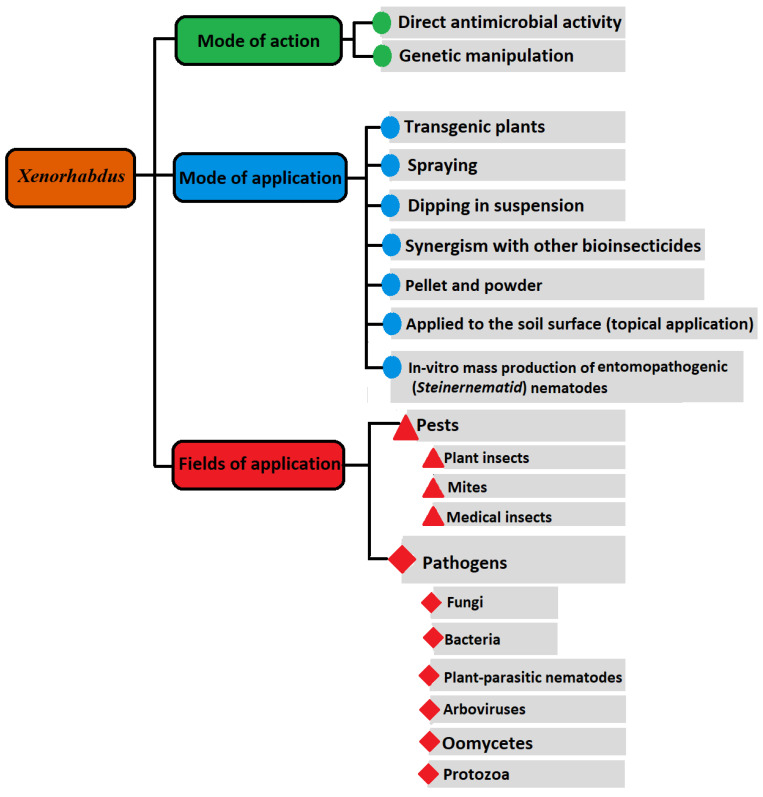
A sketch of possible framework of *Xenorhabdus* bacterial application, mode of application, and mechanism of operation to control different groups of pathogens and pests [4,5,8,11,22,24,40,41,42,44,51,52,54,55,57,62,63,67,68,69,71,72,78,79,84,98,99,105,121,125,126,129,130,133,138,141].

Discovering the competency of *Xenorhabdus* bacteria to live in fresh water and in soil for 6 days has surely opened a new avenue with fixed timeframe for their further biocontrol usages, apart from their mutualistic *Steinernema* [48]. Consequently, various formulations and techniques (Figure 1), fundamentally comprising just the bacteria or/and bacterial metabolites, have been used [2,4,5,11,17,41,42,49,50,51,52]. In this regard, boosted pathogenicity islands of the *Xenorhabdus* chromosome, having numerous genes that encode various antibiotics, insecticidal protein toxins, enzymes, and bacteriocins, were investigated [5,41,53], and more are still to be further characterized, e.g., [22,42,49,54]. For instance, only one *Xenorhabdus* strain may generate a variety of antifungal and antibacterial compounds. Some of its compounds are active against insects, protozoa, nematodes, and cancer cells, too [41]. All tested *X*. *nematophila* strains showed insecticidal activity against representative pests of three insect orders; the cabbage white caterpillar *Pieris brassicae* (Lepidoptera: Pieridae), the mosquito larva *Aedes aegypti* (Diptera: Culicidae), and the mustard beetle *Phaedon cochleariae* (Coleoptera: Chrysomelidae) [52]. In this study and others [40,44,55,56], an important note is the variation in the abilities of different *Xenorhabdus* species/strains to kill/inhibit the growth of the intended pest or pathogen. These variations are based either on the ability of each *Xenorhabdus* species/strain to generate effective metabolites or the relative susceptibility/tolerance of the targeted pest/pathogen. These differences are found not only between *Xenorhabdus* species/strains, but surely exist to varying degrees between bacterial species/strains belonging to different genera. *Photorhabdus luminescens* showed more suppressing effect against *Plutella xylostella* (the diamondback moth) than that shown by [56]. *Xenorhabdus nematophila* and *P*. *luminescens* induced 40% and 60% mortality of *P. xylostella* pupae, with LC_50_ values of 5.5 × 10^5^ and 5 × 10^4^ cells/mL, respectively. This difference does not mean the constant superiority of *Photorhabdus* over *Xenorhabdus* bacteria. On the contrary, the cells of *X. budapestensis* strain DSM 16342, *X*. *szentirmaii* strain DSM 16338, and *P. luminescens* ssp. laumondi strain TT01 could induce 100%, 88%, and 79.3% mortality rates of the locust bean moth *Ectomyelois ceratoniae* (Lepidoptera: Pyralidae) larvae, respectively, at three days’ post-exposure [57]. In addition, the cell-free culture media of the strain DSM 16342 induced 53.7% mortality, demonstrating the existence of a robust insecticidal component generated by the strain. They recommended this strain as a promising alternative biocontrol agent against *E*. *ceratoniae* to protect pomegranate (*Punica granatum*) fruit cultivars [57] were more suppressive against second-stage juveniles of *Meloidogyne javanica* than *X*. *bovienii* where the nematode-induced mortalities were 88, 94.7, and 67.7%, respectively, at 24 h post-application. To explain the difference in nematode-mortality percentages, the authors [58] reported that metabolites with nematicidal activity in *X*. *bovienii* are different from those of *X. nematophila* and *P. luminescens*. Furthermore, among nine bacterial symbionts of EPNs, *X. nematophila* HB310 showed the best insecticidal activities against *Locusta migratoria manilensis* (Orthoptera: Acrididae)-fourth instar nymph [50]. The toxicities of its culture broth against different locust instars were examined via the leaves-tip bioassays. The LC_50_ of the strain HB310 culture broth against adult, fourth instar nymph, and second instar nymph were 6.24 × 10^5^, 4.90 × 10^5^, and 2.97 × 10^5^ CFU/mL at 96 h, respectively. In addition, the LT_50_ of its culture broth were 64.34, 52.59, and 51.48 h, respectively. Moreover, this strain exerted antifeeding activity to locust where the median antifeedant concentrations (AFC50) of its culture broth were 3.16 × 10^5^, 2.36 × 10^5^, and 1.15 × 10^5^ CFU/mL at 48 h, respectively. The authors [50] concluded that strain HB310 culture broth has high and fast insecticidal activities; it could be used as insecticidal agent against the locust. Apparently, some *Xenorhabdus* species could be more effective against pests/pathogens than others related to the same genus. The adults of *Drosophila melanogaster* showed resistance to *X. innexi* [59] which was also ineffective in the death of *Manduca sexta* larvae, while *X*. *nematophila* proved effective against the two insect species [60].

An obvious technique to circumvent the lack of appropriate efficacy of *Xenorhabdus* species/strain and/or to increase its potency is to introduce other antagonists in combination with *Xenorhabdus* bacteria and/or their bioactive compounds. This approach can establish and boost the efficacy of the introduced organism, too. Clearly, synergistic activity to kill the beet army worm *Spodoptera exigua* could be obtained by mixing growth media supernatants of *Xenorhabdus* bacteria with *B. cereus* or *B. thuringiensis* spores. In this case, while the supernatant of *Xenorhabdus* bacteria could exert its impact on the insect hemocoel, the *Bacillus* cells were able to perforate the insect midgut epithelium [61,62]. Later, Eom et al. [62] could develop a “dual Bt-plus” product by mixing *B. thuringiensis* (Bt) spores and culture broth of *X. nematophila* (Xn). Although this product demonstrated high toxicity, it has also some modification to widen its efficacy against a diverse insect pest spectrum. Their tests centered on increasing “Bt-Plus” toxicity against a semi-susceptible insect, *S*. *exigua,* via adding Xn metabolites. Given the fact that Xn metabolites, benzylideneacetone (BZA) and oxindole (OI), can boost the Bt insecticidal activities, adding each of them (OI or BZA) could significantly enhance Bt-Plus pathogenicity. Moreover, when the freeze-dried Xn culture broth was included into Bt-Plus, a much smaller amount could suffice to raise the toxicity relative to the amount of BZA or OI. High-performance liquid chromatography analysis revealed that there were more than 12 unidentified *X*. *nematophila* metabolites in Xn culture broth. Therefore, they [62] proposed that there are other potent biological response modifiers in *X*. *nematophila* metabolites, not solely OI and BZA. Likewise, a *Xenorhabdus* species could induce high mortality of *S. exigua* third-instar larvae but its pathogenicity was much less for the fifth-instar larvae. Seongchae and Yonggyun [63] speculated that the high mortalities in the third-instar larvae were due to antibiotic activity against *B. cereus*, a gut symbiont needed to optimize *S. exigua* development. To enhance the *Xenorhabdus* species pathogenicity in the fifth instar, the bacteria should be delivered into the hemocoel. Thus, the authors utilized *B. thuringiensis aizawai* (*Bt*) as a synergist to back entry of the bacteria from the insect gut lumen into its hemocoel by disrupting the *S. exigua* gut epithelium. As a result, the applied bacterial mixture was highly synergistic against the *S. exigua* fifth-instar larvae. This synergism was proved via the successful infection of *X*. sp. or *Bt* in the insect hemocoel. Therefore, *Xenorhabdus* bacteria can be used to kill *S. exigua* by oral treatment in a mixture with *Bt* [63].

In other investigations, other insecticidal toxic proteins were purified from *Xenorhabdus* bacteria and/or heterologously expressed in other bacterial systems [11,64,65]. In this respect, to conquer some of their limitations, *Xenorhabdus* can be genetically engineered to enhance the potential virulence of its nematode host against more resistant herbivore pests [40]. Differently, expression of a *X. bovienii*-derived protease inhibitor protein in tobacco (*Nicotiana tobacum* cv. Samsun NN) plants could increase tolerance against green peach aphid *Myzus persicae* (Hemiptera: Aphididae). Fusion proteins of a protease inhibitor from *X*. *bovienii* strain PIN1 and green fluorescent protein (GFP) were expressed in the plant [11]. The effect of genetic transformation on anti-pest activity for *M*. *persicae* was examined by feeding neonate aphids on three independent homozygous lines. When nymphs were fed on PIN1-GFP-expressing plants, no impacts on insect survival were noticed, but fecundity and average insect weight and were considerably decreased. The aphid biomass was reduced by 30–35% relative to those reared on control plants. The effects of PIN1 on *M*. *persicae* were tightly related to the decrease of total protease activities of whole insect extracts and the leucine aminopeptidase. Additionally, an elevation in polyphenoloxidase activity was apparent in PIN1-GFP-expressing plants. These results demonstrated that the transgenic expression of PIN1 in tobacco increased plant tolerance against aphids. The study disclosed *Xenorhabdus* bacteria as other valuable resources of protease inhibitors which can be engineered into plants for insect pest management [11].

Obviously, the aforementioned traits of the *Xenorhabdus* bacteria—such as the ability to disturb the induction of insect-derived antimicrobial peptides, adjusting and fitting to the settings, particularly under stresses, and global spread with a treasure trove of beneficial materials and compounds (e.g., [16,41,42])—are quite attractive for their multiple usage. They may be a sound ground to materialize their capability in the wide and reliable biocontrol of plant pests/pathogens. Their magnificent array of (primary and secondary) metabolites may be so effective that they are, independently, absolutely capable to suppress a wide profile of pests in many groups, particularly in the orders Lepidoptera (caterpillars, moths, and butterflies), Coleoptera (weevils and beetles), and Diptera (flies, including insects that transmit human and plant diseases) [2,7,42,52]. For instance, *X*. *nematophila* cells and their metabolic secretions are lethal to the *Galleria mellonella* larvae. The bacterial toxic secretion in broth induced 95% mortality within 4 days post-application but the *X*. *nematophila* cells brought about 93% mortality at 6 days post-application [66]. The insect mortality was better in sand substrate than filter one. Likewise, *X*. *nematophila* cells and secretion in broth showed the highest efficacy at 25 °C and 14% moisture treatments. Full insect mortality (100%) was noted at *X*. *nematophila* concentration of 4 × 10^6^ cells/mL. Only within 2 h post-application could maximum *X*. *nematophila* cells in broth (95%) penetrate into the *G. mellonella* larval body. Moreover, when the bacterial toxic secretion was dried and then dissolved either in water or broth, it also proved to be effective. On the other hand, the efficacy of bacterial toxic secretion was declined due to storage. Because the bacterial cells can enjoy a free-living lifetime and invade the insect hemocoele without their nematode partner, the authors confirmed that the *Xenorhabdus* cells, or their impactful secretions, can be utilized for pest control. Therefore, they [66] suggested that *X*. *nematophila* or its toxic secretion can be utilized as a significant component of integrated pest management (IPM) programs against *Galleria*.

Usually, re-extraction of the *Xenorhabdus* bacteria from the insect cadavers and comparison with the standard (original) culture can assure Koch’s postulates [41,66]. Although the obtained data confirmed the direct toxicity of the bacteria to definite insect species in nature, e.g., [5,52,66], particular *Xenorhabdus* bacteria may have wide host range of insect pests. For example, 122 strains of symbiotic bacteria associated with 23 EPNs were gathered from various Chinese localities [67]. These extracted strains displayed oral growth inhibition and/or insecticidal activity against the *Ostrinia furnacalis* larvae. One of the strains, however, *Xenorhabdus* sp. SY5, with determined partial toxin gene sequence, exhibited strong insecticidal activity to a variety of economically significant agricultural pests. Their species comprised *Plutella xylostella*, *Ostrinia furnacalis*, *Tenebrio molitor*, *S. exigua,* and *Mythimna separata*. The strain isolated from *Steinernema* sp. SY5 appeared to have seven purified toxins based on DEAE-52 column chromatography. These toxins exhibited, to a certain extent, growth inhibition and/or insecticidal activity to these insect species. The authors [67] stressed the high virulence of this strain as a potential asset for biological pest control.

It is likely that the arsenal of *Xenorhabdus* spp. still possesses much that has not been discovered yet, for controlling wide categories of many pathogens. In this respect, Hajihassani et al. [68] assessed the efficacy of application timing, that is, 5 days before planting (DBP) and at planting (AP) of *X. bovienii* and *X. szentirmaii* metabolites for the root-knot nematode (RKN) *Meloidogyne incognita* control on cabbage roots in two environmental conditions. At-plant applications of *Paecilomyces lilacinus* strain 251 (MeloCon WG) and secondary metabolites of *Burkholderia rinojensis* strain A396 (Majestene) and oxamyl (Vydate) were used for comparison. In the greenhouse, *X. szentirmaii* and Vydate at 5 DBP had a lower (*p* < 0.05) root gall rating than the untreated control. Vydate and all metabolite treatments showed significantly lower root galling relative to Majestene, MeloCon, and the control. In addition, the metabolites and Vydate decreased (*p* < 0.05) RKN egg counts per gram of root compared to the other treatments in the greenhouse. No differences were observed in the egg count between Vydate and the metabolites. At-plant and 5 DBP applications of *X. bovienii* and *X. szentirmaii* at decreased the total egg count relative to Majestene and the control in the greenhouse. Thus, the natural metabolites generated by the two *Xenorhabdus* species can control *M*. *incognita* regardless of application timing and are suggested as a potential alternative to nematicides in organic production systems [68]. In addition, direct effect of *X. lircayensis*, identified using the whole genome, was evaluated on a population of the plant-parasitic nematode *Xiphinema index* [69]. Supernatants of bacteria were discarded via centrifugation, then *X*. *lircayensis* were resuspended in phosphate-buffered saline (PBS) and set to 1 × 10^6^ and 1 × 10^7^ CFU mL^−1^ for laboratory and semi-field assays, respectively. Cell bacteria (1 × 10^7^ CFU mL^−1^) were applied in the semi-field assay by 30 min dipping grapevine roots in the bacterial suspension. Afterward, these plants were established in 5 L pots filled with naturally *X*. *index*-infested soil and immediately inoculated with 350 mL of the same *X*. *lircayensis* suspension. The nematicidal effects of *X*. *lircayensis* suspension appeared at 24 h post-inoculation but attained full (100%) *X. index* mortality after 72 h exposition (*p* < 0.001) in laboratory assays. In addition, under semi-field conditions, *X*. *lircayensis* suspension significantly (*p* ≤ 0.05) reduced *X. index* populations. While the study recommended *X*. *lircayensis* as a good candidate for further assesses in field conditions, additional analyses must be performed to set the metabolites, enzymes, and mode of action for its nematicidal aptitude [69]. Vicente-Díez et al. [70] tested the antibiotic impact of cell-free supernatants (CFSs) and unfiltered ferments (UFs) of *X. nematophila* and *P. laumondii* on another plant pathogenic category represented by the fungus *Botrytis cinerea* growth and compared the activity of bacteria isolated from a bio-fermenter with the commercial *B. amyloliquefaciens* (Serenade^®^ASO, Bayern CropScience). The UF and CFS of *X. nematophila* suppressed about 95% and 80% of *B*. *cinerea* growth, respectively, while both UF and CFS of *P. laumondii* inhibited only about 40%. These data showed the potential of CFS and UF of *X. nematophila* for *B. cinerea* control.

In another study [71], *X. bovienii* metabolite treatment was comparable to fenbuconazole (a commercial fungicide) in decreasing *Fusicladium effusum* sporulation on pecan (*Carya illinoinensis*) terminals. *X. bovienii* metabolite and broth treatments suppressed development of lesions brought about by *Phytophthora cactorum* (using pecan tree leaves maintained on agar). The bacterial metabolite treatment was also toxic to *Armillaria tabescens*, another important pathogen but of peach (*Prunus persica*) trees, especially in the southeastern United States [71]. These results offer a basis for further investigations on utilizing the bacterial metabolites or broth for suppression of economically significant diseases of pecan and peach. Likewise, *X*. *nematophila* generates many metabolites during growth and multiplication. Only one of these secondary metabolites (xenocoumacin 1) proved to have a robust antifungal activity for controlling *Rhizoctonia solani* [72], *Botrytis cinerea* [73], *Alternaria alternate* [74], several *Phytophthora* species, etc. [73,74,75,76]. These effects suggest, a priori, that other *Xenorhabdus* species, which are available or are likely to befit broadly soon, are able to control other pests and diseases. Recently, *Xenorhabdus budapestensis* strain C72 showed remarkable suppressing effect on spore germination and mycelial growth of the fungus *Bipolaris maydis* which causes the Southern corn leaf blight [77]. The relative control effect of the bacterial cell-free culture media reached 59.15% and 77.96% in greenhouse and field experiments, respectively, which was as efficacious as a commercial fungicide. The in vitro tests also indicated that C72 cell-free culture media with thermostability proved wide-spectrum antifungal efficacy towards other economically significant fungi and pathogens of plants [77]. Chacón-Orozco et al. [78] reported that among 16 strains of EPN-symbiotic bacteria, cell-free supernatants of *X*. *szentrimaii* had the highest fungicidal effect on mycelium growth of *Sclerotinia sclerotiorum*. They reported that *X*. *szentrimaii* produces volatile organic compounds that inhibit *S*. *sclerotiorum* growth and/or its consequent generation of sclerotia.

The discovery and cloning of additional useful compounds from *Xenorhabdus* are still in progress [1,22,43,54,57]. Factually, these bacteria can demonstrate metabolites with the major characteristics of safe pesticides. In other words, their effect is boosted with an enhanced dose, but a negative correlation is found between the number or density of pathogen/pest eggs, adult survival of the pest, percentage of hatching, and the *Xenorhabdus* bacterial dosage [5,41,42].

### 3.2. Xenorhabdus Bacterial Mechanism via Their Secreted Materials

The *Xenorhabdus* bacteria are typically famous for killing their hosts via toxemia/septicemia, within the form of the normal *Xenorhabdus*–*Steinernema* complex [5]. However, as different *Steinernema* species carrying specific *Xenorhabdus* strains can invade a single insect, *Xenorhabdus* spp. are also engaged in competition with both related strains and nonrelated gut microbes of the insect host [79]. This competition, in addition to *Xenorhabdus* having the capability to kill the insect host, can explain why *Xenorhabdus* spp. produce a treasure trove of diverse insecticidal and antimicrobial compounds. Moreover, Ciezki [79] found that *X*. *bovienii* and *X*. *nematophila* can generate R-type bacteriocins (xenorhabdicins) that are specifically active towards different *Xenorhabdus* and *Photorhabdus* species. The latter author stressed that xenorhabdicin activity could be predictive of competitive results between two *Xenorhabdus* strains, while other determinants, besides xenorhabdicins, were mainly included in the competitive success between the other *Xenorhabdus* strains. Thus, Ciezki [79] demonstrated that various *Xenorhabdus* antibiotics could define the output of interspecies competition in a natural host environment.

The mounting ambition to harness *Xenorhabdus*-derived compounds in industrial products stems from not only their abundance, but also their qualities that enhance their functions. Initially, standalone pathogenicity trials of *Xenorhabdus* bacteria and/or their released materials usually start with their direct injection into the haemocoel of insects via artificial means [5,66]. *Xenorhabdus* protein toxins ordinarily have oral or/and injectable toxicity to insects. *Xenorhabdus*-derived compounds have a variety of modes of action that have been reviewed [41,69,79]. The suggested mode of action of *Xenorhabdus*–dithiopyrrolone derivatives (comprising the two metabolites xenorhabdins and xenorxides) is inhibition of RNA synthesis [80]. However, *Xenorhabdus*–indole-containing compounds could show additional mechanism via weak phospholipase A2 inhibitory effects. This latter, phospholipase A2, is necessary for producing eicosanoids. Eicosanoids have substantial role for activating the insect-immune response via mediating and modulating hemocyte behavior [81]. Thus, Dreyer et al. [41] assumed that indole-containing materials produced by *X*. *nematophila* can suppress the immune response of the insect host to be more vulnerable to microbial infection. *Xenorhabdus* *budapestensis* has two antimicrobial peptides, GP-19 and EP-20, with wide-spectrum antimicrobial activity against bacteria and fungi [82]. The first peptide, with a neutral charge, is suggested to cause a disruptive impact to the host membrane by moving to the cell surface and penetrating the membrane. The second peptide likely has a different mode of action. It is suggested to have an intracellular influence, by inhibiting protein synthesis, cell wall, and nucleic acid [82].

Complete genome sequencing of various *Xenorhabdus* species/strains has been uncovering the ability of these bacteria to produce numerous secondary metabolites. Thus, it can contribute to comprehensive examination of the molecular basis underlying the biological control activity of this *Xenorhabdus* strain [83]. Various types of biological molecules have been detected and characterized for *Xenorhabdus* bacteria. The main antimicrobial materials comprise ribosomal-encoded benzylideneace-tone [84] xenocin and bicornutin [85,86], and non-ribosomally generated xenematides [87], fabclavines [88], xenocoumacin [89], nematophin [90], rhabdopeptides [91], and peptide–antimicrobial–*Xenorhabdus* lipopeptides [92]. Knowing the attributes of these compounds, e.g., the range of pH and heating needed for their stability, should enable their successful use as alternatives to chemical pesticides in agriculture [40,41]. For example, depsipeptides are peptides that generally have alternating peptide and ester bonds, and five classes of depsipeptides have been characterized. The first class, produced by *Xenorhabdus doucetiae* and *X. mauleonii* and known as xenoamicin, are tridecadepsipeptides with hydrophobic amino acids [93]. The genome sequence of *X. doucetiae* DSM 17909 revealed that xenoamicins are encoded by five non-ribosomal peptide synthetases (NRPSs), XabABCD, and an aspartic acid decarboxylase (XabE). Due to its hydrophobic characteristics, xenoamicin can interact with the host–cytoplasmic membrane. Nevertheless, no antifungal or antibacterial activity has been listed for xenoamicin A, which displays a different mechanism. Xenoamicin A has weak cytotoxic and anti-protozoal activities [93]. The second class of depsipeptides, the lipodepsipeptides produced by *X. indica*, has supplemental fatty acid chain linked to one of the amino acids [94]. The peptides are named after their amino acid sequence and are known as taxlllaids (A–G). Natural taxlllaid A and synthetic taxlllaids B–G can manifest antiprotozoal activity. Taxlllaid A is optimistically cytotoxic to human carcinoma cells [94]. The third depsipeptides class are grouped as indole-containing xenematides. Xenematide A, secreted by *X*. *nematophila* [95], is antibacterial and insecticidal. The other two depsipeptide classes contain szentiamide and xenobactin isolated from *X*. *szentirmaii* and *Xenorhabdus* sp., strain PB30.3 [96,97]. Both compounds are active against *Plasmodium falciparum* (protozoan parasite of humans) and have some activity against *Trypanosoma brucei rhodesiense* and *Trypanosoma cruzi* (parasites of many vertebrates). Szentiamide possesses a weak cytotoxic activity against *Galleria mellonella* hemocytes. Xenobactin has no cytotoxic activity; yet, it is active against *Micrococcus luteus*. This antibacterial activity is mostly due to its hydrophobic status where it probably targets the bacterial cell membrane [40]. Eventually, each of the aforementioned groups of toxins has a conceivable role as a biocontrol material, via a particular mode of action against pathogens and arthropod pests such as vector insects. The differential virulence of the candidate toxins can be correlated not only with their interspecies/strain gene sequence diversity of the same EPN-symbiotic bacterial genus but also between the two EPN-symbiotic bacterial genera, *Xenorhabdus* and *Photorhabdus* [51,78]. Fabclavine is broadly generated in *Xenorhabdus* species but *Photorhabdus* species do not produce fabclavines, except for *P. asymbiotica* [98]. This can elucidate partially why the tested *Photorhabdus* species (*P*. *kayaii*, *P*. *namnaoensis*, *P*. *laumondii*, *P*. *akhurstii*, *P*. *thracensis*) did not show antiprotozoal activity [51]. On the contrary, fabclavines 1a and 1b demonstrate diverse bioactivities against various bacterial, fungal, and protozoal organisms [89]. Other antiprotozoal bioactive materials produced by 22 *Xenorhabdus* species are xenorhabdins, xenocoumacins, and PAX peptides. Thus, the tested *Xenorhabdus* species were more effective against the serious human protozoal parasites *Entamoeba histolytica*, *Acanthamoeba castellanii*, *Trichomonas vaginalis*, *Trypanosoma cruzi*, and *Leishmania tropica* [51]. Furthermore, it is quite possible that more *Xenorhabdus*-derived toxins will uncover certain variations among bacterial strains regarding toxicity to these pests. Various features and details concerning the mode of action, structure, and putative function of the *Xenorhabdus*-bioactive compounds in the process of infection have been clarified [41,42,79].

While many detected metabolic compounds of *Xenorhabdus* bacteria with their beneficial functions are known (Table 1), the modes of action of their other compounds are still required to be understood, to facilitate their sound utilization in the management of agricultural pests/pathogens [8,51]. *Xenorhabdus* bacteria can control economically significant endoparasitic species of nematodes inside plant roots via their antibiotic compounds and toxins [99]. Moreover, the bacterium-derived protease inhibitor protein could be genetically transformed into tobacco plants in order to offer protection from the aphids *Myzus persicae* [11]. Therefore, such genetically engineered techniques are suggested as a promising replacement to the Bt toxin [18], to preclude development of insect resistance [1]. The numerous instances of pathogen and arthropod pest killing induced by *Xenorhabdus* spp. [5,22,42,46,54,82,99] do not deny the variations in the immune response among their pathogen/pest hosts [6,22,51,52]. In addition, the difference in immune reaction among host species/strains may be based on biologic/genetic and evolutionary/ecological factors set for each pathogen–host system. The various system constituents, including specificity, induction, and memory of the immunity, can determine the cognate resistance mechanism of the intended insect population/species [100]. Generally, physical parameters, especially pH, temperature, and sodium chloride, could variably affect the mortality percentage induced by these metabolites to the *G*. *mellonella* larvae [101].

## 4. The Positive and Negative Aspects of *Xenorhabdus* spp.

### 4.1. Major and Direct Favorable Aspects

The growing interest in investigating *Xenorhabdus* bacteria is justified by major testimonies available in the literature, to name but a few: (i) possessing genes that are responsible for encoding low-molecular-weight secondary metabolites/toxins with various activities against many pests/pathogens, e.g., insects [17,42], fungi [78,79], protozoa [49,51], bacteria [41,80], plant-parasitic nematodes (PPNs) [70,121], and other parasites [22,122]; (ii) many research tests indicate the success of *Xenorhabdus* bacteria in pest control under laboratory conditions [52,123] with some experimentations showing promise in large crop production areas (e.g., [99]) and under field conditions (e.g., [124]); (iii) *Xenorhabdus bacteria* releases toxins with substantial activities in the insect intestinal epithelium [5,125]; (iv) *Xenorhabdus* bacteria can have synergistic activity, when added to other biocontrol agents, against serious and intractable insect pests such as the beet army worm [62,63]; (v) specific bacterial proteins could be effective against certain insect pests, e.g., *X*. *ehlersii* protein (XeGroEL) is reliable for *G. mellonella* control [126,127]; (vi) toxins and metabolites of certain *Xenorhabdus* bacteria could have acaricidal and antibacterial activities, e.g., *X*. *stockiae* PB09 [128,129]; (vii) the aforementioned *Xenorhabdus*-derived bioactive compounds can offer novel templates for commercial use, obtaining reliable and environmentally safe alternatives to currently unsafe pesticides, e.g., to control phytonematodes [54] and mites [130]; (viii) *Xenorhabdus nematophila*-derived bioactive compounds blocked the feeding of crickets, ants, and wasps [131,132]; (ix) commercial pesticides could operate more effectively via advantageous inputs of specific *Xenorhabdus*-derived metabolites such as oxindole and benzylideneacetone [63]. *Xenorhabdus*-derived bioactive compounds could be used not only in agricultural sustainable systems but also in pharmaceutical and industrial products. A mixture of *Bacillus thuringiensis israelensis* (*Bti)* spores with *X. nematophila* culture broth containing metabolites has superior efficacy in controlling *Aedes albopictus* mosquitoes and *Culex pipiens pallens*. Based on enhanced *Bti* toxicity in the mixture against the two insect species, a commercial product was created, named “Dip-Kill” [133]. The culture broths of *P. temperate temperata* and *X. hominickii* could also boost the toxicity of *Bti* against culicides [2,133].

### 4.2. Favorable Aspects That Need Further Exploration

Several published books and tomes, e.g., [134,135,136], of EPNs and their symbionts mostly focus on their original use as insecticides. Hence, other avenues for other applications of *Xenorhabdus* bacteria against additional pests and pathogens should be further explored. For instance, *Xenorhabdus* bacteria proved useful against serious PPN species [69,70,99,121]. *X. bovienii* played a key role in decreasing PPN populations in turfgrass [137]. Notably, dipping the tomato roots into *X*. *bovienii* supernatant immediately before planting to infested soil was the most effective treatment for both *M*. *arenaria* and *M*. *incognita* control compared to treatments based on EPN-infected insect cadaver formulations using different EPN species, EPN IJs, and the cell-free supernatants of their mutualistic bacteria grown in liquid culture [121]. The treatment with *X*. *bovienii* supernatant considerably decreased numbers of RKN egg masses, but increased plant height and enhanced fresh and dry weights relative to the infested control plants. Later, to further test and improve the materials tested by Kepenekci et al. [121], the seedling roots were dipped into 192-hour-old *X*. *bovienii* bacterial supernatant just before tomato transplanting to the soil. Another bacterial treatment included applying 10 mL *X*. *bovienii* supernatant to the soil surface [99]. The two bacterial treatments were compared to others using *Steinernema feltiae*–IJs, their infected insect cadavers, and the nematode-parasitic fungus *Purpureocillium lilacinum* in addition to the RKN-infested check plants. In their greenhouse-2 test in the Kepez region, Turkey, they found that the highest (2.94 ± 0.10 kg tomato fruit/plant) and lowest (2.11 ± 0.09 kg tomato fruit/plant) were harvested from *X. bovienii* (dipped + topical application) treatment and control, respectively (*p* < 0.0001). While such results furnish growers with hands-on knowledge for decision-making to determine the financial feasibility of these different RKN control strategies [99], they provide researchers and stakeholders with data to further optimize relevant biocontrol techniques and avoid their inconsistent effects. The authors [99] speculated that such applications of *Xenorhabdus* bacteria can suppress infection by plant pathogenic fungi [40], in addition to reducing RKN populations. Thus, they assumed that what was measured [99] could sometimes be an underestimate of the value of applying the symbiotic bacteria. Further exploration of *Xenorhabdus* bacterial efficacy on various pests and pathogens associated with tomato, or other PPNs-susceptible plant species, in specific agricultural systems should lead to conclusive and practical results.

Tomato plants expressing certain genes of *X. nematophila* gained not only resistance to the cotton bollworm *Helicoverpa armigera* (Lepidoptera: Noctuidae) but also tolerance against salt and thermal stresses [138]. With such multiple benefits, reassessing social and economic effects^1^ of genetically engineered crops is challenging and requires further investigations. Such crops may be introduced to growers in rural communities with varying social communities and heterogeneous agricultural systems. On the other hand, there is a great potential of using *Xenorhabdus* bacteria in pharmaceutical and industrial products. For example, their products can be used to control vector insects of human diseases such as dengue and malaria. In this respect, occurrence of mosquito’s resistance to *Bti* was documented [2]. Nonetheless, mixing the bacteria with *Xenorhabdus nematophila*-cultured broth could deter the resistance and strengthen toxicity against such vector insects as *Aedes albopictus* and *Culex pipiens pallens* (Diptera: Culicidae) [2,133]. Further research should be directed for using *Xenorhabdus* bacteria against mosquito-borne arboviruses and other microorganisms of the *Plasmodium* group.

### 4.3. Cost-Effective Xenorhabdus Mass Culture, Formulation, and Application

Contrary to *P. luminescens*, cell phenotypic variation in *X*. *nematophila* strains could be controlled via regular selection of primary variants. Consequently, no trait change was detected in the *X*. *nematophila* primary variant after prolonged subculture. The genetic basis for this stability is a merit for the bacterial economical and biocontrol applications of *X*. *nematophila* production [139] because the tested *Xenorhabdus* bacteria have superior production (i.e., reduced culture time and scale-up) against the need to preserve traits significant for biocontrol. Furthermore, the fermentation process was recently optimized for xenocoumacin 1 (Xcn 1) production by *X*. *nematophila* [75,140]. As Xcn 1 has excellent activity against bacteria, oomycetes, and fungi, the obtained 243.38% increase in Xcn1 production [140] can lay a foundation for its industrial production. In another study, the *X*. *nematophila* fermentation production of Xcn1 was raised to 3.4-fold (234.9 mg/L) and 3.6-fold (249.7 mg/L) at 1.0 and 0.5% L-arabinose concentration, respectively [75]. These improvements should be extended to other *Xenorhabdus*-bioactive compounds.

Various *Xenorhabdus* formulations have been implemented (Figure 1) with specific examples representing varying degrees of successful and failed/uneconomical formulation and application. Among successful trials, *X*. *stockiae* PB09 formulated in wettable powder or liquid cell pellet could economically induce high miticidal mortalities of 90.25, 86.50, and 92.78% for the mushroom mite (*Luciaphorus perniciosus*). These formulations have potential to be further developed as commercial products for controlling mushroom mites [130]. An opposite example that still has major obstacles to beat before implementation is the use of *X.* s*zentirmaii* in a powder form against the fungus *Botrytis* *cinerea*. This effect of the antifungal metabolites of *X.* s*zentirmaii* in a powder form was weaker than its corresponding liquid *X.* s*zentirmaii* supernatant [141]. Because *Xenorhabdus* powder has longer shelf-life and can be easily and economically formulated and applied compared to liquid supernatants, the author suggested that further studies should optimize the processes for the powder formulation to enhance its efficacy. In such a case, the system should possess all factors for success including high levels of *Xenorhabdus* virulence, favorable pulverization process, proper contact with the tested fungal pathogen, and compatibility with current practices, e.g., temperature.

### 4.4. Other Aspects of Xenorhabdus spp.

Contrary to *Photorhabdus* bacteria, which has the human-pathogenic *P*. *asymbiotica* [5], *Xenorhabdus* bacteria have never been detected from clinical specimens [5]. However, with the exception of some studies in phylogenetics and molecular identifications, *Xenorhabdus* bacterial studies on their bioactive compounds have somewhat lagged behind those of *Photorhabdus* bacteria. For instance, the high-molecular-weight insecticidal “toxin complexes” were first identified in *P. luminescens* strain W14 and subsequently in *X. nematophila* [142]. To conclude this review, it suffices that this article referred to the above examples that clearly express their counterparts, leaving more relevant details in their original references. It is needless to remind the reader that using these bacteria singly or with their host nematodes are mostly costly and may comprise sometimes inconsistent results due to many biological and ecological factors that should be specified to suit the used biocontrol agents [143]. Yet, the changing scope of crop protection necessitates facing such challenges for sustainable agriculture [144].

## 5. Conclusions

Accelerated public concerns over the misuse and impact of chemical pesticides on human health and environment create the need to discover alternative methods for management of plant pests and pathogens. Utilizing *Xenorhabdus* bacteria as biologicals in various sustainable agricultural systems against a wide range of plant pests and pathogens is apparently an attractive and promising approach. This bacterium can be exploited directly (with its nematode partner) or indirectly (without its partner) to neutralize the plant–phytonematode interaction to the advantage of the plant. Such an emerging approach should be tried in earnest in order to control not only PPNs, but also many other plant pests and pathogens within IPM plans. The detection of new *Xenorhabdus* species and strains globally will occur to widen the pool of these bacterial-related materials that are reliable for managing economically significant pathogens/pests. Developing low-priced techniques for their trade production, formulation, and application may expedite their use in current or future management programs. In such programs, *Xenorhabdus* bacteria can be used with their *Steinernema* host or as standalone pesticides for common management strategies. Obviously, these bacteria produce a treasure trove of diverse compounds that can kill many harmful insects and microorganisms, e.g., protozoa, PPNs, fungi, oomycetes, and bacteria. *Xenorhabdus* bacteria could be applied in various formats for topical application, e.g., pellets, powder, spray, suspension, or supernatant. Furthermore, their toxin genes could be incorporated into transgenic plants to control insect pests. The transgenic plants could also gain tolerance against salt and thermal stress in some cases. Therefore, *Xenorhabdus* bacteria and their bioactive compounds are highlighted herein; in order to provide a substantial way forward in pest management and crop protection, they should be leveraged to fit into holistic crop management strategies. These may comprise their combination with other additive or synergistic inputs, to enhance their efficacy while blocking, or decreasing, the likelihood of developing pesticide-resistant harmful strains.

## Figures and Tables

**Table 1:** Bioactive compounds generated by *Xenorhabdus* bacteria *.

*Bacterial* spp.	Bioactive Complexes/ Secondary Metabolite	Biological Asset	References
*X. beddingii*	R-type bacteriocins	Bactericidal	[102]
*X. bovienii*	Xenocyloins	Insecticidal	[103]
	Amicoumacin	Antibacterial, insecticidal, antifungal, anticancer, and anti-inflammatory	[104]
	Indoles	Antibiotic	[105]
	Dithiolopyrrolones	Antibiotic	[105]
*X. budapestensis*	Bicornitun	Antibacterial and antifungal	[98]
	GP-19	Antibacterial and antifungal	[83]
	EP-20	Antifungal	
	Fabclavine	Antibacterial, antifungal, antiprotozoal, and cytotoxic	[106]
*X. cabanillasii*	Nemaucin	Antibacterial and antifungal	[107]
	Rhabdopeptide	Antiprotozoal, insecticidal, and cytotoxic	[108]
	Cabanillasin	Antifungal	[109]
*X* *. doucetiae*	Xenoamicin	Antiprotozoal	[110]
	Xenorhabdin	Antibacterial	
	Xenocoumacin	Antibacterial, antifungal, and antiulcer	
*X. ehlersii*	XeGroEL protein	Insecticidal	[42]
*X. indica*	Taxlllaids	Antiprotozoal and cytotoxic	[95]
*X* *. kozodoii*	Xenocoumacin	Antibacterial, antifungal, and antiulcer	[98]
*Xenorhabdus* *spp.*	Xenobactin	Antibacterial and antiprotozoal	[97]
*X. khoisanae* *strain SB10*	PAX lipopeptides	Antimicrobial	[43]
	Xenocoumacin	Antimicrobial	[43]
*X. innexi*	Rhabdopeptides	Antiprotozoal, insecticidal, and cytotoxic	[111]
*X* *. mauleonii*	Xenoamicin	Antiprotozoal	[98]
	xenocoumacin	Antibacterial, antifungal, and antiulcer	
	xenorhabdin	Antibacterial	
*X. nematophila*	Pristinamycin	Antibacterial	[112]
	Xenorhabdins	Antibacterial	[113]
	Xenorxides	Antibacterial and antifungal	
	PAX peptides	Antibacterial and antifungal	[40]
	Nematophin	Antibacterial and antifungal	[114]
	Xenocin	Antibacterial	[87]
	Xenorhabdicin (R-type bacteriocins)	Antibacterial	[44]
	Xenocoumacins	Antibacterial, antifungal, and antiulcer	[115]
	Xenortides	Antiprotozoal and cytotoxic	[116]
	Rhabdopeptides	Antiprotozoal, insecticidal, and cytotoxic	[92]
	Xenematides	Antibacterial and insecticidal	[117]
	Rhabducin	Insecticidal	[117]
	Oxindole and Benzylidene-acetone	Antibacterial, immuno-suppressant, and insecticidal	[41,85]
*X. szentirmaii*	Fabclavines	Antibacterial, antifungal, antiprotozoal, and cytotoxic	[106]
	Szentiamide and Xenocin (peptide xcinA sequenced)	Antibacterial, antifungal, and/or cytotoxicity	[41,118]
	Xenofuranones	A and B Insecticidal	[119]
	Xenocoumacin	Antimicrobial	[55]
	PAX lipopeptides	Antimicrobial	[55]
	Iodinin (1,6-dihydroxyphenazine 5,10-dioxide)	Antimicrobial multidrug-resistant pathogens	[120]

* Compiled from recent references [22,41,42].

## Data Availability

Not applicable.
